# Thermostabilisation of the neurotensin receptor NTS1

**DOI:** 10.1016/j.jmb.2009.04.068

**Published:** 2009-05-05

**Authors:** Yoko Shibata, Jim F. White, Maria J. Serrano-Vega, Francesca Magnani, Amanda L. Aloia, Reinhard Grisshammer, Christopher G. Tate

**Affiliations:** 1 MRC Laboratory of Molecular Biology, Hills Road, Cambridge CB2 0QH, UK; 2 Membrane Protein Structure and Function Unit, National Institute of Neurological Disorders and Stroke, National Institutes of Health, Department of Health and Human Services, Rockville, MD 20852, USA

**Keywords:** membrane protein, G protein-coupled receptor, conformational thermostabilisation

## Abstract

Structural studies on G protein-coupled receptors (GPCRs) have been hampered for many years by their instability in detergent solution and by the number of potential conformations that receptors can adopt. Recently, the structures of the β_1_ and β_2_ adrenergic receptors and the adenosine A_2a_ receptor were determined with antagonist bound, a receptor conformation that is thought to be more stable than the agonist-bound state. In contrast to these receptors, the neurotensin receptor NTS1 is much less stable in detergent solution. We have therefore used a systematic mutational approach coupled to activity assays to identify receptor mutants suitable for crystallisation, both alone and in complex with the peptide agonist, neurotensin. The best receptor mutant, NTS1-7m, contained 4 point mutations. It showed increased stability compared to the wild type receptor, in the absence of ligand, after solubilisation with a variety of detergents. In addition, NTS1-7m bound to neurotensin was more stable than unliganded NTS1-7m. Of the four thermostabilising mutations, only one residue (A86L) is predicted to be in the lipid environment. In contrast, I260A appears to be buried within the transmembrane helix bundle, F342A may form a distant part of the putative ligand binding site, whereas F358A is likely to be in a region important for receptor activation. NTS1-7m binds neurotensin with a similar affinity to the wild-type receptor. However, agonist dissociation was slower, and NTS1-7m activated G proteins poorly. The affinity of NTS1-7m for the antagonist SR48692 was also lower than that of the wild-type receptor. Thus we have successfully stabilised NTS1 in an agonist-binding conformation that does not efficiently couple to G proteins.

## Introduction

Determination of membrane protein structures has long been regarded as a difficult area of structural biology. To obtain diffraction-quality crystals, a target membrane protein needs to be available in sufficient quantities, be stable in detergent solution from which crystallisation occurs, and be obtainable in one particular conformation. Stability in a range of detergents is central to crystal formation, but other factors such as the removal of flexible protein parts or the choice of the crystallisation system (vapour diffusion or lipid based approaches) may also need to be considered 1. Increasing successes with bacterial membrane proteins, such as transporters and ion channels, have shown that many of the perceived difficulties in membrane protein crystallisation can now be overcome. The first membrane protein structures solved were all rigid, stable proteins and came from natural sources: a photosynthetic reaction centre^2^ and an outer membrane porin^3^ from bacteria, and the bovine cytochrome c oxidase^4^ and bc1 complex^5^, from mitochondria. More recently, eukaryotic membrane proteins from heterologously expressed sources have also been crystallised *e.g.* the rat Kv1.2 voltage-gated potassium channel expressed in *Pichia pastoris*^6^ and a chicken acid-sensing ion channel expressed in insect cells^7^.

One commonality between the eukaryotic membrane proteins, whose structures have been solved, may be their relative stability in detergent, a consideration that also applies to many prokaryotic membrane proteins. Unfortunately, a direct comparison of the stability for all the membrane proteins in detergents in which they have been crystallised has not been carried out. However, many successful crystallisation conditions, especially for eukaryotic membrane proteins, included ligands, lipids, and/or lipid-like compounds, with the aim of improving the stability of a particular membrane protein during crystallisation. If stability in detergent is one of the key determinants of crystallisability, then solving structures of a large number of human membrane proteins may currently not be possible, because solubilising these proteins in detergent would inactivate them.

Attempts to determine the structures of G protein-coupled receptors (GPCRs) have been ongoing for over 20 years. Bovine rhodopsin was the first GPCR to be crystallised^8^; ^9^; ^10^, reflecting its relatively high stability in many detergents as long as the receptor was kept in its inactive, dark state. Many other GPCRs have been over-produced in a variety of expression systems^11^, and some of them have been purified to homogeneity. However, it has only been recently that structures have been determined for the human β_2_-adrenergic receptor (β_2_AR)^12^; ^13^ and the human adenosine A_2a_ receptor (A_2a_R)^14^ using the T4 lysozyme fusion strategy and lipidic cubic phase crystallisation procedures, whilst the structure of a thermostabilised β_1_-adrenergic receptor (β_1_AR) was determined from crystals grown in detergent by vapour diffusion^15^. The β_2_AR was also crystallised in complex with an antibody fragment using the bicelle system^16^. These recent successes are due partly to increased usage of microfocus beamlines to collect x-ray diffraction data from very small crystals, but also to our understanding of how to maintain receptors in a biologically relevant, single conformation, long enough for crystallisation to occur. This was achieved by inclusion, during crystallisation, of high-affinity antagonists/inverse agonists (β_2_AR, β_1_AR, A_2a_R) or cholesteryl hemisuccinate (β_2_AR, A_2a_R), all of which are predicted to improve stability of the receptor during crystallisation^14^; ^17^; ^18^; ^19^; ^20^. Receptor stability has also been improved by site-directed mutagenesis (β_1_AR), which allowed the use of more denaturing short-chain detergents for crystallisation^19^. Insertion of the T4 lysozyme fusion partner in the flexible third cytoplasmic loop of receptors appears to improve the crystallisability of the receptor (β_2_AR, A_2a_R)^12^; ^14^, although the effect of T4 lysozyme on the stability of the receptor is unknown.

We now know the structures of 5 GPCRs in an inactive, antagonist-bound state (β_2_AR, β_1_AR, A_2a_R, bovine rhodopsin, and squid rhodopsin^21^) and the structure of one GPCR, bovine opsin, in an active-like state^22^; ^23^. Although the structures of antagonist-bound GPCRs show great similarities within the transmembrane cores, there are also differences, especially in the conformations of the extracellular and intracellular loops, such that it is not yet possible to predict in atomic detail how, for example, a specific ligand binds to a receptor of unknown structure. The β_1_AR, β_2_AR and A_2a_R bind small ligands within the transmembrane core and their structures in the antagonist-bound states are similar to the structure of dark-state rhodopsin. However, larger ligands such as peptides involve receptor regions other than the transmembrane bundle for binding and agonist-bound receptors, regardless of the sizes of their ligands, are thought to be much more flexible and/or to undergo rapid equilibrium between multiple structural states^24^. Therefore the agonist-bound activated states of peptide receptors are still unknown territory in GPCR structures.

We have focussed our attention on a peptide receptor, the neurotensin receptor, NTS1^25^. Neurotensin is a 13 amino acid peptide agonist that is thought to bind in an extended conformation^26^ to the receptor at a site formed by both extracellular loops and transmembrane helices (TM) as predicted from mutagenesis and structure-activity studies combined with modelling techniques^27^; ^28^. The agonist binding site overlaps with that of the antagonist SR48692, although the small synthetic antagonist requires only the TM helices for its binding^29^. Extensive work on the expression and purification of NTS1 has led to the production of milligram quantities of highly-purified functional receptor from *Escherichia coli*^30^ but diffraction quality crystals have not yet been obtained. NTS1 is not particularly stable in detergent; it requires the presence of cholesteryl hemisuccinate and glycerol throughout the purification to retain ligand-binding activity, and its stability in short-chain detergents is severely compromised. We therefore decided to identify thermostabilising NTS1 mutants to allow the use of a wider range of detergents and buffer conditions for crystallisation. Such a mutagenesis approach has successfully identified mutations in both bacterial membrane proteins^31^; ^32^ and GPCRs^18^; ^19^, and conformational thermostabilisation of the β_1_AR in an antagonist-bound form^18^ was essential for its subsequent structure determination at 2.7 Å resolution^15^. One constraint that we imposed upon the stabilisation procedure was that NTS1 needed to be stabilised ideally in both the unliganded- and neurotensin-bound states, because this would allow the rapid purification of the mutated receptor using a well-established automated procedure^30^. Here we describe the successful thermostabilisation of NTS1 in both the unliganded- and neurotensin-bound states.

## Results

### Development of thermostability assays for the neurotensin receptor

**Figure 1:**
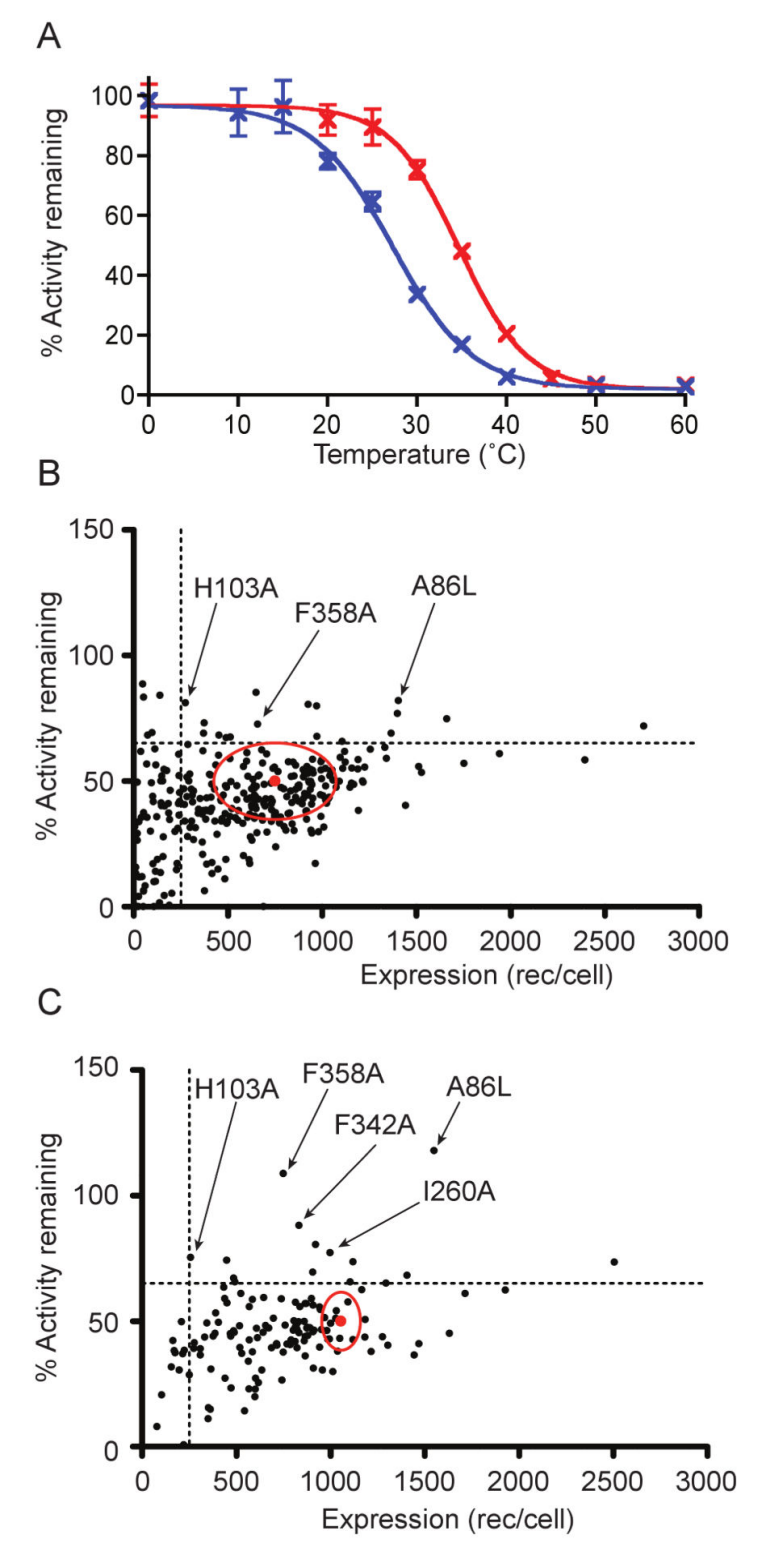
Thermal stability and expression levels of NTS1 single Ala/Leu mutants. (A) The thermal stability of wt-NTS1 in the unliganded state (blue) and with neurotensin bound (red) was assessed by determining the apparent T_m_ value from the mid-point of the curves; apparent T_m_ of unliganded wt-NTS1, 24±2°C; apparent T_m_ of neurotensin-bound wt-NTS1, 37±2°C. (B-C) Individual mutants of NTS1, each containing a single alanine mutation (if the original amino acid was alanine then it was mutated to leucine) are summarized for its expression level in *E. coli* (number of functional receptors/cell), its thermal stability in the absence of neurotensin (B), and in the presence of neurotensin (C). Thermal stability was measured after incubating each detergent-solubilized mutant at 24 °C (B) or 37 °C (C) for 30 minutes, and the percentage of activity remaining after incubation was determined with respect to its own unheated control. All the stability data are normalized against the wt-NTS1 stability for each set of experiment (wt=50 %). The mean wt-NTS1 expression level and stability (red dot) and standard errors (red oval) are shown in the plots. The dotted lines show the cut-off values for the stabilized mutants (65 % activity remaining, 250 receptors/cell).

An essential prerequisite to our thermostabilisation strategy is to develop a robust thermostability assay for the unpurified detergent-solubilised receptor based upon radioligand binding^18^; ^19^. In this instance, the thermostability of NTS1 was determined using a [^3^H]-neurotensin ([^3^H]-NT) binding assay. To directly compare the stability of wild-type neurotensin receptor (wt-NTS1) with that of β_1_AR, wt-NTS1 was solubilised and its thermostability determined in a buffer system similar to that used for β_1_AR, containing only DDM^19^. However, the apparent T_m_ in DDM was rather low with limited reproducibility, possibly because solubilised unliganded wt-NTS1 was too unstable and therefore was sensitive to fluctuations in the laboratory temperature (results not shown). To improve the reproducibility of the thermostability assays, CHAPS, CHS and glycerol, with final concentrations of 0.6 %, 0.12 % and 30 %, respectively, were included during the solubilisation in 1 % DDM, conditions which had previously been found to be highly stabilising^33^. The concentration of NaCl in the thermostability assay was kept as low as possible (27 mM in the assay buffer, carried over from the lysis buffer), as a high concentration of Na^+^ ions is known to inhibit NT binding to the receptor^34^; ^35^. The concentration of [^3^H]-NT used in the assays (12 nM) was at least 5-fold above the apparent K_D_ value for detergent-solubilised wt-NTS1 (K_D_ 1-2 nM in this buffer condition; results not shown) to allow high receptor occupancy, but which kept non-specific [^3^H]-NT binding to a minimum. Under these conditions, wt-NTS1 showed an apparent T_m_ of 24±2 °C in the unliganded state, and 37±2 °C with NT bound (Fig. 1A). The apparent T_m_ was defined as the temperature at which 50 % of solubilised receptor remained functional after incubation for 30 minutes.

**Figure 2:**
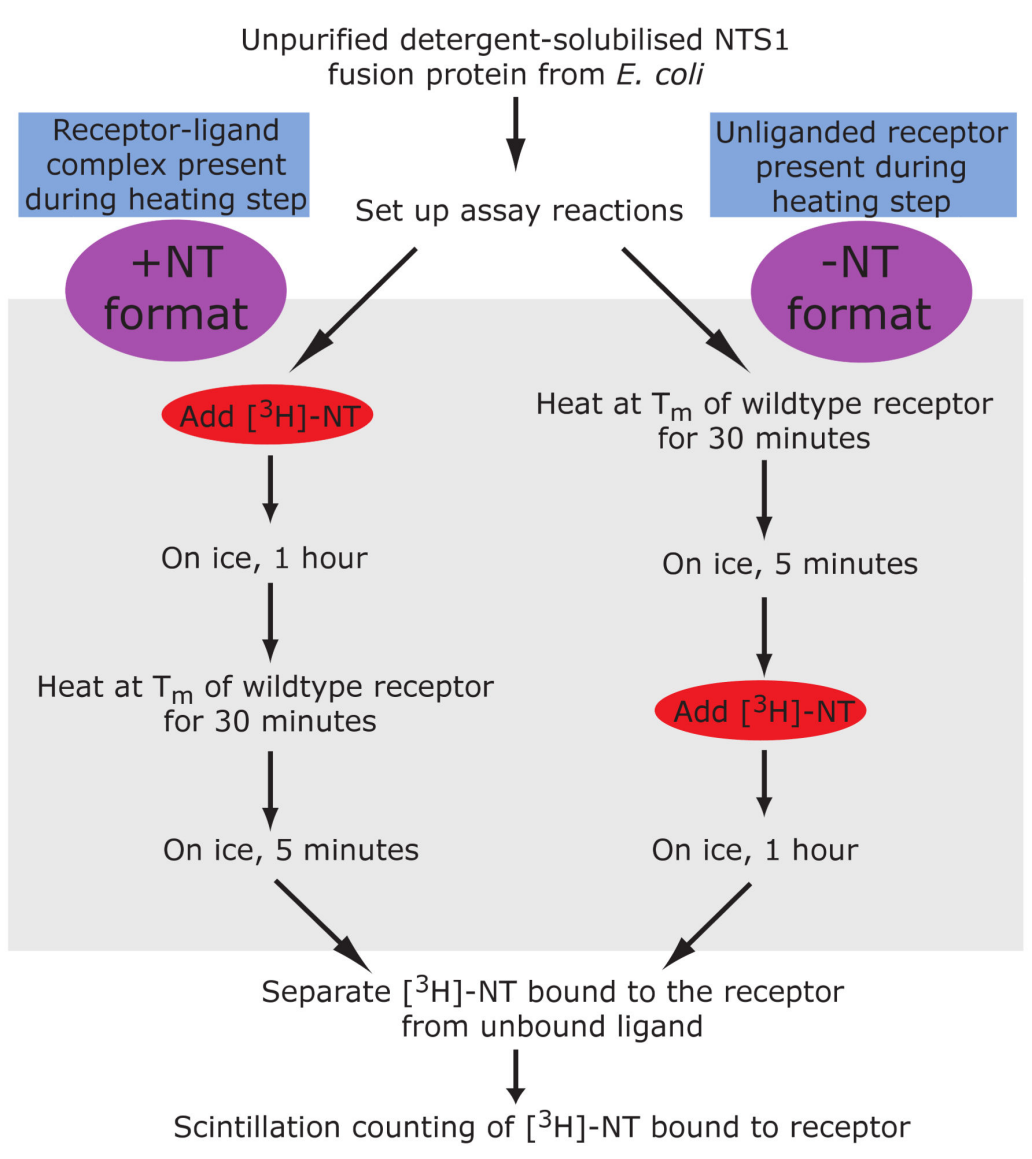
Schematic of the +NT and −NT thermostability assays.

In designing the thermostabilisation strategy for NTS1, we also considered how the receptor was going to be purified and the most likely state in which we would want to crystallise it. Crystallisation is best performed under conditions where the receptor is most stable; NTS1 should therefore be crystallised with NT bound as suggested by the thermostability assay (Fig. 1A). However, the purification scheme for NTS1 relies upon a ligand affinity purification step using a NT-affinity column^30^. Therefore, the ideal NTS1 construct for structural studies would be stable in both the presence and absence of NT. With this in mind, we developed two thermostability assay formats for detergent-solubilised NTS1, which we refer to as the “-NT assay” and the “+NT assay” (Fig. 2). In the -NT assay, solubilised NTS1 was heated at the apparent T_m_ without ligand (24 °C; Fig. 1A) for 30 minutes, placed on ice, [^3^H]-NT was then added and, after a 1 hour incubation on ice, the amount of [^3^H]-NT bound to the receptor was determined by using a mini-gel filtration spin column to separate the receptor-ligand complex from free [^3^H]-NT. In the +NT assay, the 30-minute heating step at 37 °C (apparent T_m_ with NT bound; Fig. 1A) was performed after the addition of [^3^H]-NT. Thus the -NT assay determined the stability of the unliganded receptor and the +NT assay determined the stability of the NT-bound NTS1.

### Screening Ala/Leu scan mutants for thermostability in the unliganded state

**Figure 3:**
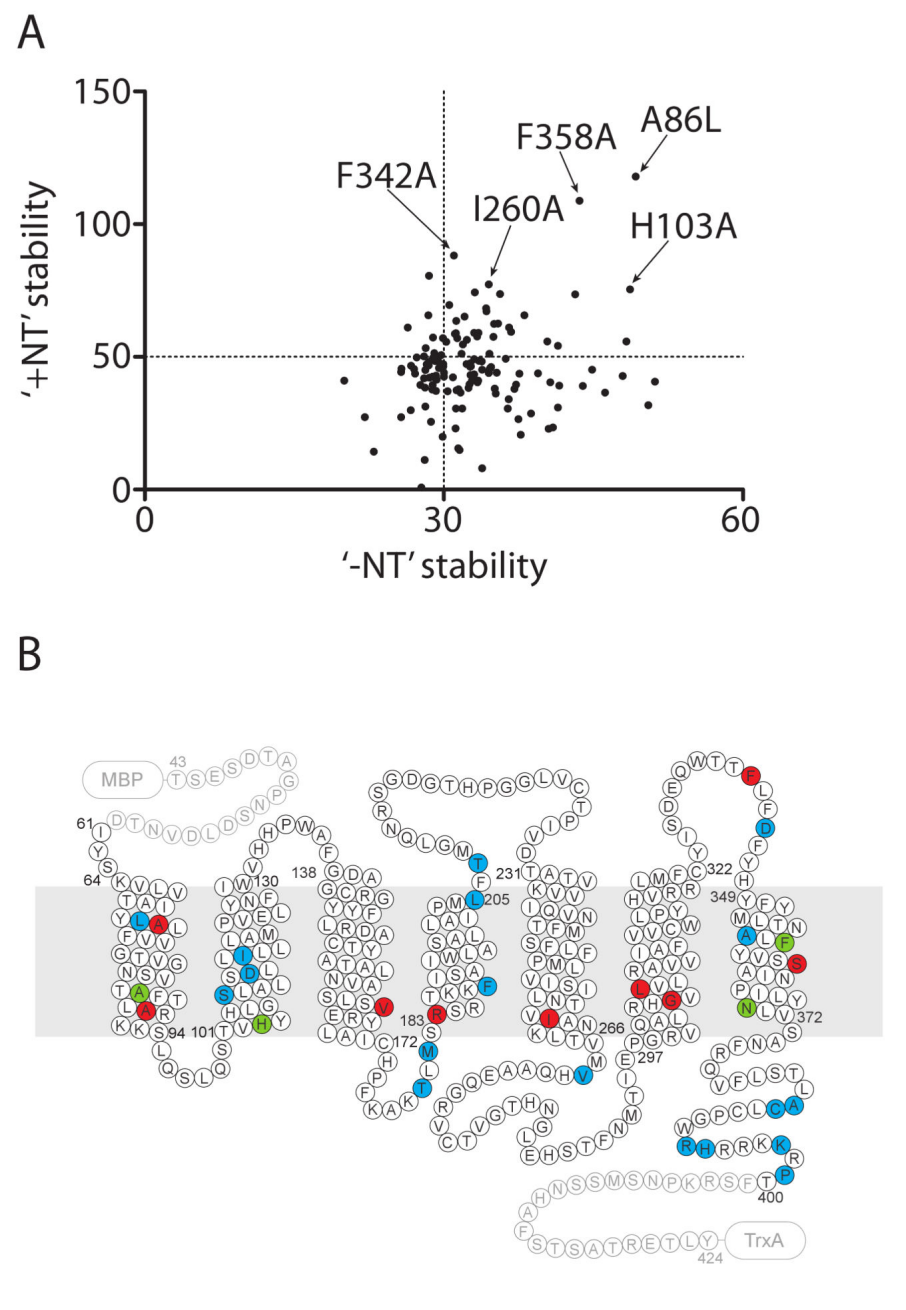
Comparison of unliganded-state and agonist-bound state stabilities of NTS1 single Ala/Leu mutants. (A) The stability of each mutant is shown in both the unliganded state (-NT stability) and agonist-bound state (+NT stability). Mutations combined to optimally stabilise NTS1 are indicated. The intersection of the dotted lines in the plot corresponds to the position of wt-NTS1. (B) The locations of 31 stabilizing mutations are shown in the snake plot; positions of stabilising mutations are shown for the unliganded receptor (blue), neurotensin-bound receptor (red) or both (green).

We made 340 point mutations throughout NTS1 from Ile^61^ to Thr^400^ and expressed them as maltose-binding protein (MBP) fusions in *E. coli.* Three hundred and eleven positions were mutated to alanine, and the 29 native alanine residues were changed to leucine. Western blots of whole cell lysates probed with anti-MBP antibody showed similar intensities of bands corresponding to the NTS1 fusion protein (within three fold of wild-type expression level) for all the mutants except for C142A and C225A. These cysteine residues are predicted to form a disulfide bond, and mutating either one of them led to a dramatic reduction in expression levels of the full-length fusion proteins and increased proteolysis (results not shown). [^3^H]-NT ligand binding assays (LBA) performed on detergent-solubilised receptors at 4 °C revealed that 50 mutants did not bind agonist, including C142A and C225A, possibly because the respective NTS1 mutants were misfolded or because a given mutation reduced directly or indirectly the agonist affinity such that [^3^H]-NT binding could not be detected at the ligand concentration used in the assay. Thermostability assays were performed on each of the 290 functional mutants. The assays were performed first in the -NT assay format to determine the stability of unliganded detergent-solubilised NTS1 mutants. The percentage of remaining functional receptor was determined by comparing the amount of bound [^3^H]-NT after heating with its unheated control. As the percentage of active wt-NTS1 remaining after heating varied between 30-50 %, the mutant values were scaled to values expected if the wt-NTS1 activity remaining was 50 %, thus allowing direct comparison between different batches of data. The accumulated error estimated for the activity assays, when expressed as number of receptors per cell, was ±15 %. In summary, out of all 340 NTS1 mutants constructed, 22 (6 %) showed an improvement in stability *i.e.* greater than 65 % functional receptors remaining after the incubation for 30 min at 24 °C (Fig. 1B). In contrast, 201 mutants (59 %) retained similar stability to wt-NTS1 in the unliganded state (∼35 % to 65 % activity remaining after heating), 67 mutants (20 %) were less stable than wt-NTS1, and 50 mutants (15 %) did not bind neurotensin (as mentioned above). The positions of the 22 best thermostabilising mutations of NTS1 in the absence of ligand (Fig. 3B, blue and green) neither conform to an obvious pattern nor do they correspond to the locations of thermostabilising mutations identified in β_1_AR^19^ or A_2a_R^18^.

Most of the thermostabilised mutants retained at least 50 % of the total number of functionally expressed receptors per cell compared to wt-NTS1 (Fig. 1B). However, we also observed exceptions. For example, the mutation H103A stabilised the unliganded receptor by 7-8 °C, but expression of H103A was nearly 4-fold lower than that of wt-NTS1, making this mutant less attractive for further use. In order to improve the expression level, five other amino acid residues were used to substitute this residue, namely H103N, H103S, H103V, H103L and H103M. Of these five mutants, H103N and H103S expressed at the same level as wt-NTS1, whilst H103V showed only a slight improvement in expression over H103A; all 3 mutations maintained the thermostability conferred by H103A. In contrast, H103L and H103M neither regained the expression level of wt-NTS1, nor retained the thermostability of H103A (data not shown). Although it was not necessary to use any of these additional changes in subsequent mutants, these data show that the low expression level of a functional, thermostable receptor can be improved by changing the thermostabilising mutation to different amino acid residues.

The best thermostabilising mutations of the NTS1 unliganded state for combination into an optimally stable receptor were chosen only after considering their effect upon NTS1 expression. We considered that 250 functional receptors/*E. coli* cell (about 25 % of wt-NTS1 expression) was a minimum acceptable level of expression and, using this as our cut off, 19 out of 22 NTS1 mutants were retained for the subsequent study. Each of these stabilizing mutations gave an increase in the apparent T_m_ value in the unliganded state of 2-10 °C, compared to wt-NTS1 (SI Table 1).

### Re-screening the Ala/Leu scan mutants for thermostability in the NT-bound state

**Figure 4:**
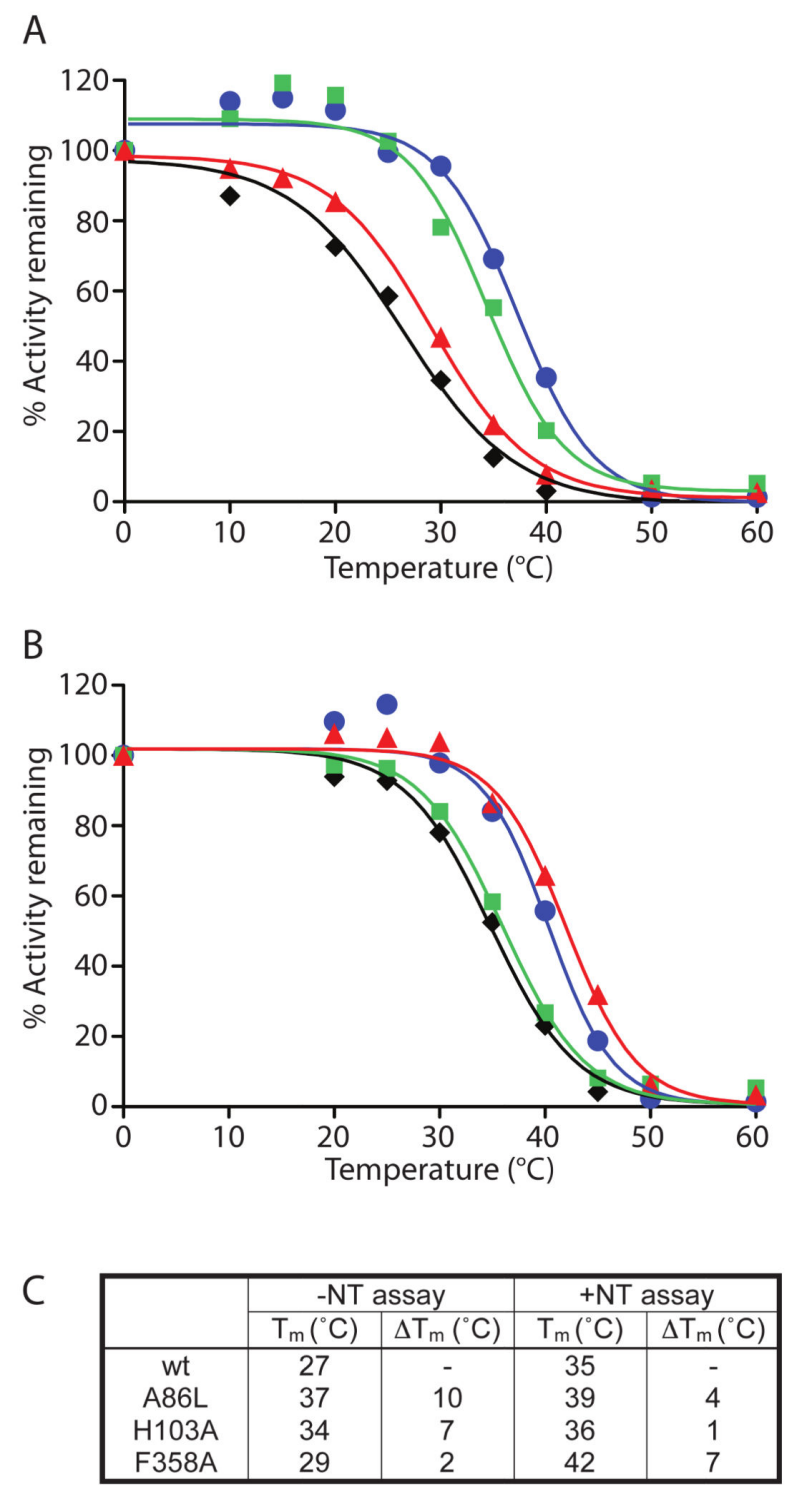
Denaturation profiles of the three best NTS1 single Ala/Leu mutants in the absence and presence of neurotensin. Denaturation curves of the three best thermostable single mutants of NTS1, A86L, H103A, and F358A, were determined by heating the solubilized mutants at elevated temperatures for 30 minutes, either in the absence of neurotensin (A) or in the presence of 12 nM [^3^H]-NT (B). NTS1 mutants shown are: wt (black diamonds), A86L (blue circles), H103A (green squares), and F358A (red triangles). (C) Table summarizing the apparent T_m_ values determined by non-linear regression of the above curves; constraint of upper lower boundaries was not used. The estimated error from repeated experiments is ±2 °C. Activity remaining was normalised to 100% based upon the amount of binding measured in the samples incubated on ice.

All 22 thermostabilising Ala/Leu single mutants selected in the unliganded state (-NT assay) were subsequently tested for thermostability in the neurotensin-bound state (+NT assay). To our surprise, many of the mutants were less stable than wt-NTS1 when bound to NT, although they were clearly more stable than wt-NTS1 in the unliganded state (not shown). In fact, 7 out of the 22 mutants were less stable in the +NT assay than wt-NTS1 (SI Table 1). Therefore, we decided to re-analyse the mutants that retained >50 % activity, *i.e.* at least as stable as wt-NTS1, in the -NT assay. Thus 137 mutants were detergent solubilised and heated in the presence of [^3^H]-NT at 37 °C for 30 minutes (+NT assay; Fig. 2). Only 13 mutants out of 137 screened were found to be more stable than wt-NTS1 in the NT-bound state (cut off value set to ∼65 %). There appeared to be little correlation between the stabilities of the mutants in the unliganded-state and the NT-bound state (Fig. 3A) and there was no discernable pattern from the positions of the mutations in the primary amino acid sequence (Fig. 3B, red and green). The 13 mutants chosen by the +NT assay were, from the selection strategy we imposed, more stable than wt-NTS1 in the NT-bound state and at least as stable as wt-NTS1 in unliganded state, and 11 out of the 13 mutants also expressed reasonably well (SI Table 1). Each of the stabilising mutations had apparent T_m_s of 1-7 °C higher than wt-NTS1 in the NT-bound state. Only 4 mutants were more stable than wt-NTS1 in both the unliganded and NT-bound states, of which only three mutants were significantly more stable, namely A86L, H103A, and F358A (Fig. 3B, green). Denaturation profiles of these three mutants and of wt-NTS1 are shown in Fig. 4, in the absence (Fig. 4A) and in the presence (Fig. 4B) of bound [^3^H]-NT and the apparent T_m_ values are shown in Fig. 4C.

### Combining mutations to further improve receptor stability

**Figure 5:**
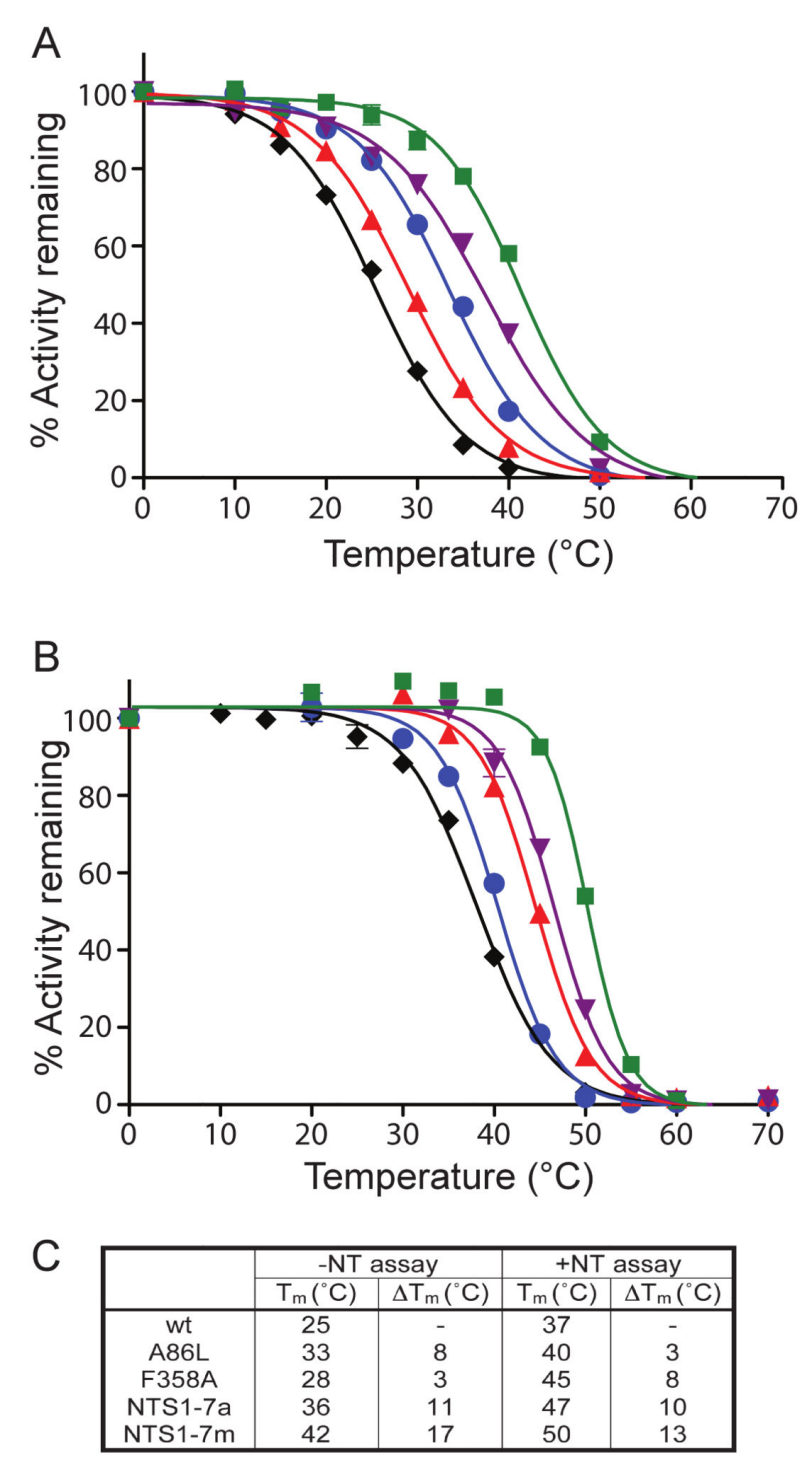
Denaturation profiles of NTS1 multiple mutants in the absence or presence of neurotensin. Denaturation curves of four examples of the best thermostable mutants, NTS1-7a (A86L/F358A), NTS1-7m (A86L/I260A/F342A/F358A), A86L and F358A were compared to wt-NTS1. The solubilized receptors were heated for 30 minutes, either in the absence (A) or presence (B) of neurotensin: wt-NTS1 (black diamonds), A86L (blue closed circles), F358A (red triangles), NTS1-7a (purple diamonds), and NTS1-7m (green squares). (C) Table summarizing the apparent T_m_ values determined from the above curves. The estimated error from repeated experiments is ±2 °C. Activity remaining was normalised to 100% based upon the amount of binding measured in the samples incubated on ice.

To evolve an NTS1 mutant that was stable both in the presence and absence of NT, we chose 14 single mutations that stabilised the unliganded state (from the -NT assay) and another 13 mutations that stabilised the NT-bound state (from the +NT assay), including the 4 mutations that appeared in both groups. Mutants that were significantly less stable than wt-NTS1 in the NT-bound state, *e.g.* L72A, and/or had low expression levels, *e.g.* D345A (SI Table 1), were not used. Combinations within the subsets of mutations were obtained by PCR using random mixtures of primers as previously described for the thermostabilisation of β_1_AR^19^ and A_2a_R^18^. The most thermostable mutants contained combinations of the five mutations, A86L, H103A, I260A, F342A and F358A (SI Table 2). Most of these mutants also maintained reasonably good expression levels, with many showing improved levels of expression over wt-NTS1. The assay conditions had to be revised at this point by increasing the incubation temperatures (to 37 °C for the ‘-NT’ assay, to 47 °C for the ‘+NT’ assay) to ensure better differentiation in the degree of stabilisation between mutants. Under these conditions wt-NTS1 showed no binding activity, so results were normalised to A86L which retained about 20 % of its initial activity in both assay formats (Fig. 5). Among the 26 mutant combinations tested, six combinations showed improved thermostability in the absence of NT (score >30), and 11 in the presence of NT (score >80) over the respective stability of A86L (SI Table 2). Mutants containing both A86L and F358A mutations appeared to show the best stabilization effects in both screening conditions. NTS1-7a, which only contains two mutations (A86L and F358A) gave more than 10 °C stabilisation compared to wt-NTS1 in both the +/-NT formats (Fig. 5). NTS1-7m (A86L, I260A, F342A, and F358A) was found to be one of the most thermostable mutants, with an apparent T_m_ of 50 °C in the presence of NT (13 °C better than wt-NTS1, Fig. 5) and an apparent T_m_ of 42 °C in the absence of NT (17 °C better than wt-NTS1, Fig. 5).

### Ligand binding properties of thermostable NTS1 mutants

**Figure 6:**
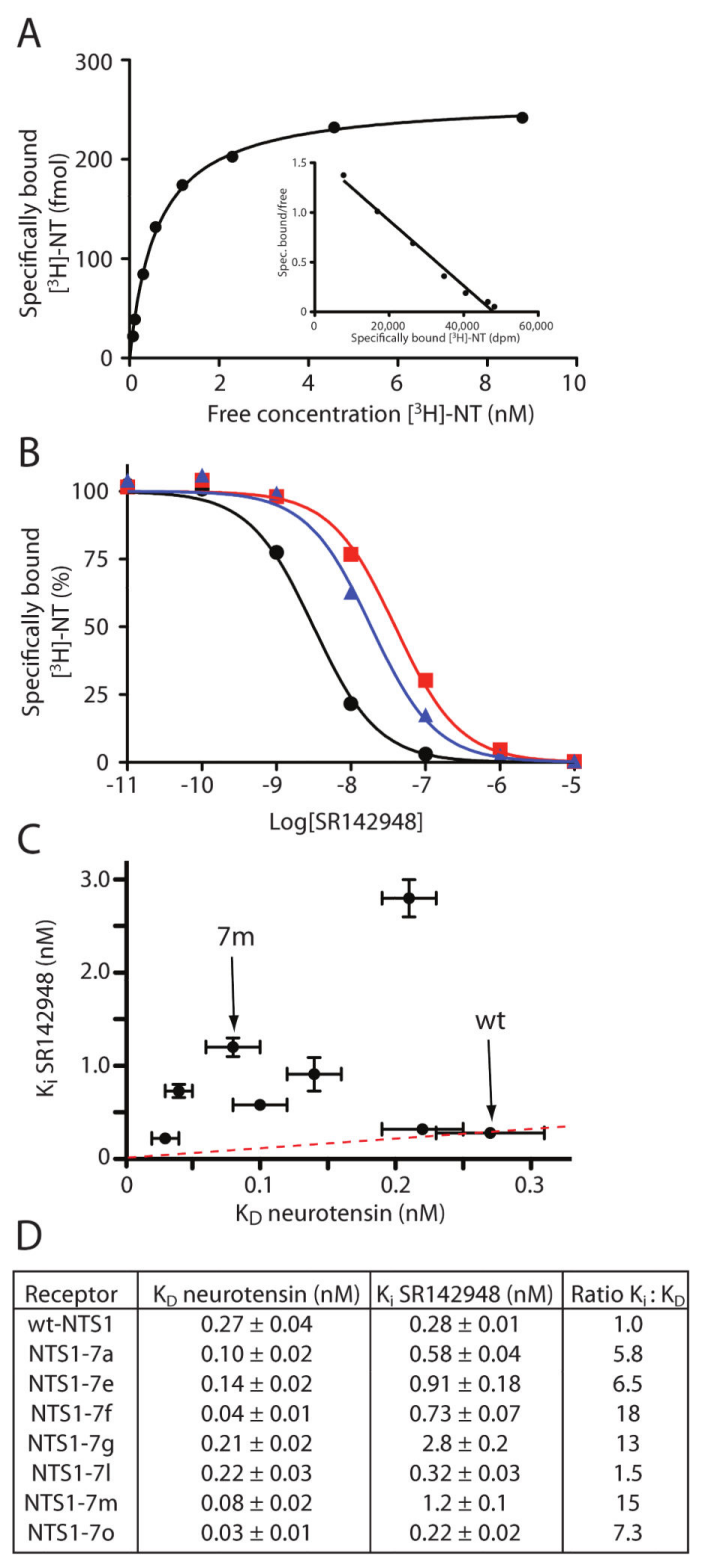
Agonist and antagonist binding to NTS1 mutants. (A) Saturation binding curve of a representative [^3^H]-NT binding experiment with NTS1-7m in intact *E. coli* cells. The Scatchard plot is shown as an inset (one-site fit, K_D_ 0.34±0.03 nM). (B) Competition assays were performed using intact *E. coli* cells expressing either wt-NTS1 or the NTS1-mutants. Increasing quantities of antagonist SR142948 were incubated with the cells in the presence of 5 nM agonist [^3^H]-NT. Competition curves for wt-NTS1 (black circles), NTS1-7a (blue triangles) and NTS1-7m (red squares) are shown. Ki values were determined by non-linear regression analyses using K_D_ values for NT-binding determined from the saturation binding curves (D). (C) The correlation between the K_D_(NT) and K_i_(SR142948) for each mutant is shown as a scatter plot with results shown of a representative experiment, with error bars representing the SEM of data fitting. The red dashed line represents the ratio between the Ki and K_D_ values for wt-NTS1. (D) Table summarizing the apparent K_D_ values for [^3^H]-NT binding, Ki values for SR142948 and the ratio K_i_:K_D_ for each of the mutants tested. K_D_ and Ki determinations were performed simultaneously for each mutant in duplicate. SEMs are from one representative experiment and arise from data fitting.

To define the effects of the mutations on the ligand binding properties of mutant receptors, the apparent K_D_s for agonist ([^3^H]-NT) binding were determined by saturation binding assays using intact *E. coli* cells expressing wt-NTS1 or selected mutants. Competition binding curves for the displacement of [^3^H]-NT by the antagonist SR142948 were used to determine K_i_ values for its binding. Seven mutants that showed good stabilisation in one or both +/-NT assays and reasonable expressions levels were tested (Fig. 6). All the thermostabilised NTS1 mutants had an apparent K_D_ for neurotensin binding either similar to that of wt-NTS1 (K_D_=0.27 nM on intact *E. coli* cells) or slightly better (Fig. 6). K_D_ values of NT for the mutants varied between 0.03 nM for NTS1-7o and 0.22 nM for NTS1-7l. In contrast, the affinities of NTS1 mutants for the antagonist SR142948 varied from near wild-type values of 0.22 nM for NTS1-7o to 2.8 nM for NTS1-7g when determined in competition with [^3^H]-NT (Fig. 6). If the ratio of K_i_(SR146948):K_D_(NT) is determined, then all the mutants tested, with the exception of NTS1-7l, showed preferential binding to NT compared to SR146948 by a factor of up to 18 for NTS1-7f.

### Characterisation of NTS1-7m

**Figure 7:**
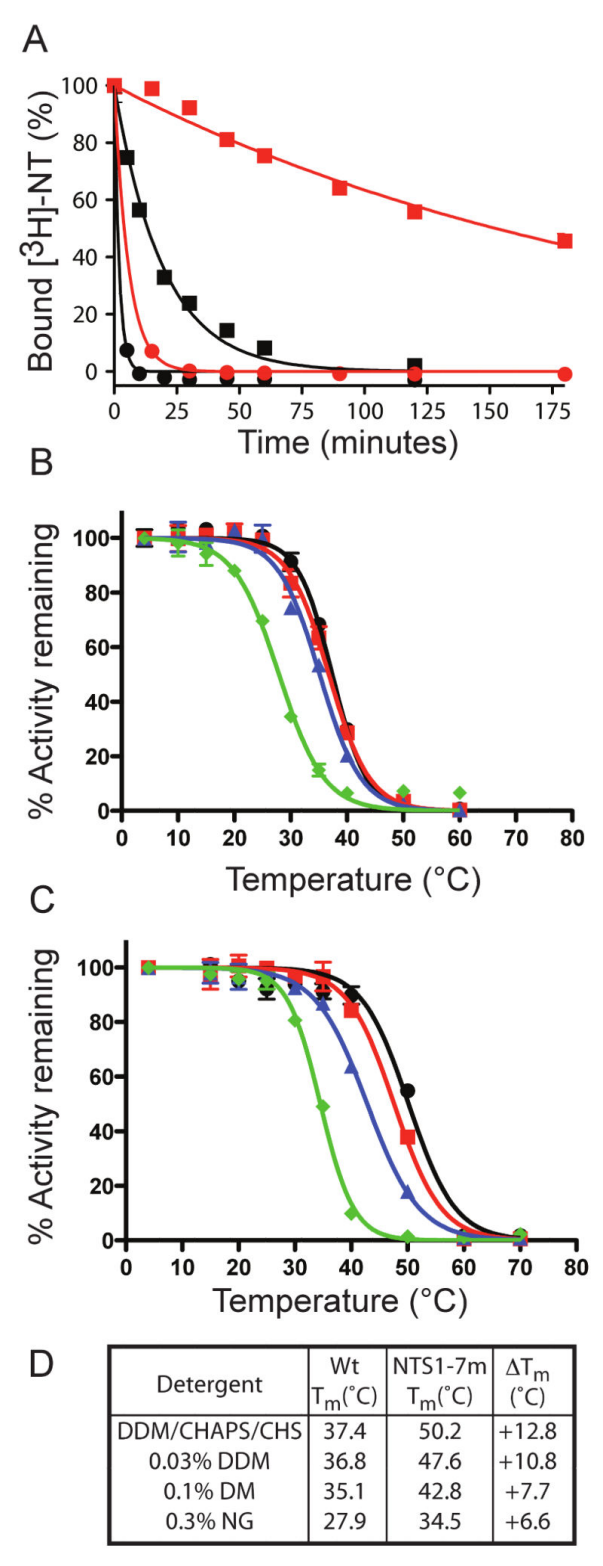
NTS1-7m shows improved thermal stability as well as stability in short-chain detergents compared to wt-NTS1. (A) The rates of thermal inactivation of solubilised wt-NTS1 (circles) and NTS1-7m (squares) in DDM/CHAPS/CHS were compared by heating the samples at 45°C either in the presence (red lines) or absence (black lines) of [^3^H]-NT. Half-lives were determined from the curves by non-linear regression of the single-exponential curve after constraining the values for Y=0 to 100 % and the plateau=0 %: unliganded wt-NTS1, 1.3 minutes; neurotensin-bound wt-NTS1, 5.7 minutes; unliganded NTS1-7m, 13.4 minutes; neurotensin-bound NTS1-7m, 220 minutes. (B-D) Thermostability of wt-NTS1 and NTS1-7m in various detergents. Receptors were solubilised in DDM/CHAPS/CHS, bound to Ni^2+^-NTA beads and then washed and eluted with buffer containing either DDM/CHAPS/CHS (black circles), 0.03 % DDM (red squares), 0.1 % DM (blue triangles) or 0.3 % NG green diamonds). Thermostability assays were performed in the presence of NT (B: wt-NTS1, C: NTS1-7m). The activity remaining was normalised against the unheated control in each detergent condition (100%), although the recovery yields were different in each case (see main text). The apparent T_m_ values (D) were determined from the curves by non-linear regression.

NTS1-7m satisfied our original aim of stabilising NTS1 in both unliganded and NT-bound states. In addition to the thermal denaturation profiles of detergent-solubilised NTS-7m (Fig. 5), we determined the degree of thermostabilisation by measuring the rate of thermal inactivation at 45 °C for detergent-solubilized wt-NTS1 and NTS1-7m (Fig. 7A). Under the conditions used, the half-lives for NTS1-7m were either 220 minutes or 13.4 minutes, in the presence or absence of bound NT, respectively, compared to values of 5.7 minutes and 1.3 minutes for wt-NTS1. Based on these half-lives, NT-bound NTS1-7m was 39-fold more stable than NT-bound wt-NTS1 and was 10-fold more stable when the unliganded receptors were compared.

Thermostabilisation of β_1_AR and A_2a_R in DDM resulted in both these receptors gaining stability in short chain detergents that are more denaturing, but are more suitable for crystallisation^18^; ^19^. We therefore tested whether NTS1-7m that was thermostabilised in DDM/CHAPS/CHS showed increased stability in other detergents that are more preferable for crystallisation. NTS1-7m and wt-NTS1 were solubilised in DDM/CHAPS/CHS, bound to Ni^2+^-affinity resin and washed either with DDM/CHAPS/CHS (the original detergent condition), 0.03 % DDM, 0.1 % DM or 0.3 % NG. The receptors were eluted in the desired detergents, and thermal denaturation profiles were determined by heating the receptors in the presence of [^3^H]-NT (Fig. 7B and C). NTS1-7m was consistently more stable than wt-NTS1 in any of the detergents tested, with apparent T_m_s 7-13°C higher than the wild-type receptor. As expected, the stability of NTS1-7m decreased as the size of the detergent micelle around the receptor also decreased, reflecting the increasing harshness of detergents (DDM<DM<NG) with shorter hydrophobic chains. The amount of functional receptor eluted from the Ni^2+^-NTA column was also consistently higher for NTS1-7m compared to wt-NTS; for example, washing and eluting of the receptors in NG recovered only 3 % of the functional wt-NTS1 compared to 20 % for NTS1-7m (compared to values determined in DDM/CHAPS/CHS). Although NTS1-7m was consistently more stable in short-chain detergents than wt-NTS1, the degree of stabilization was less than observed in DDM/CHAPS/CHS (Fig. 7D).

**Figure 8:**
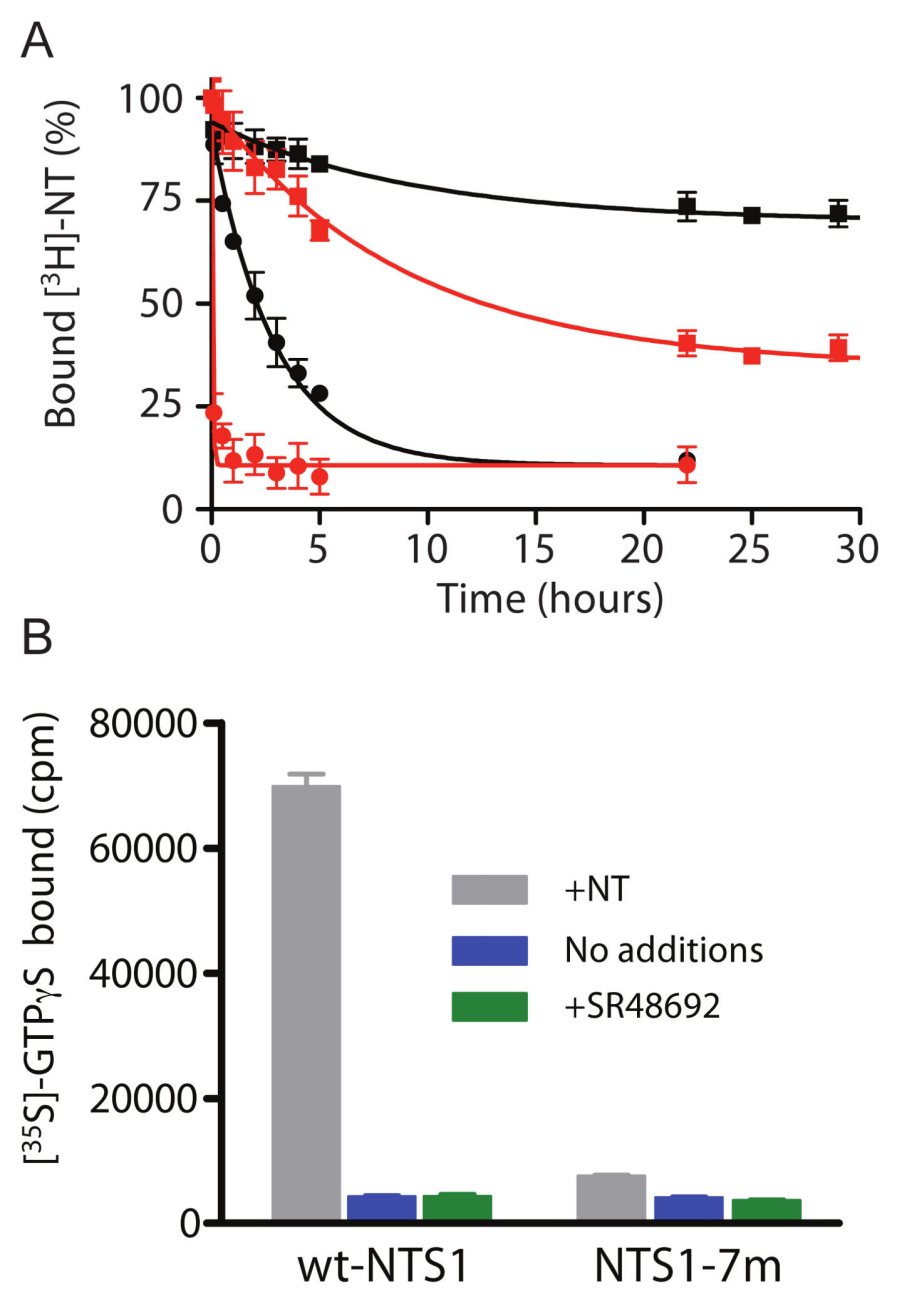
Rate of dissociation of NT and activation of G protein by wt-NTS1 and NTS1-7m. (A) The dissociation rates of [^3^H]-NT from wt-NTS1 (circles) and NTS1-7m (squares) were determined by quantifying the amount of [^3^H]-NT remaining bound to the receptors (total NT concentration in the assay, 2 nM) upon addition of 50 μM unlabeled NT on ice in the presence (red) or absence (black) of NaCl. The rate of [^3^H]-NT dissociation were determined by non-linear regression with single exponential decay. (B) Recombinant receptors in urea-washed insect cell membranes were tested for their ability to stimulate G protein using a GDP/[^35^S]-GTPγS exchange assay. All assays contained purified recombinant Gαqβ_1_γ_1_, [^35^S]-GTPγS and insect cell membranes containing either wt-NTS1 or NTS1-7m. Receptors were incubated with no additional ligands (blue bars), with neurotensin (grey bars) or with the antagonist SR48692 (green bars). The amount of [^35^S]-GTPγS bound to the G protein complex was determined as described in the text.

To investigate what might contribute to the stability of NTS1-7m, the rate of agonist dissociation from detergent-solubilised receptors (in 0.1 % DDM, 0.2 % CHAPS, and 0.04 % CHS) was determined (Fig. 8A). In this detergent condition, the K_D_ values of NTS1-7m and wt-NTS1 are 0.66 and 1.1 nM (results not shown). Although the exact K_D_ values depend on the concentrations of detergents used, the relative orders in affinity of mutant-NTS1s and wt-NTS1 are unchanged. The dissociation rate of NT from wt-NTS1 in the presence of NaCl is 50-fold higher than in the absence of the salt (Fig. 8A). On the other hand, the effect of NaCl on the dissociation rate was only ∼2-fold for NTS1-7m. Therefore, the off-rate of NT from wt-NTS1 was ∼8 fold faster than that from NTS1-7m in the absence of NaCl, but in the presence of 1M NaCl the off-rate of NT from wt-NTS1 was over 200 fold faster than from NTS1-7m (Fig. 8A).

The ability of NTS1-7m to couple to the G protein Gαq Gβ_1_γ_1_ was tested in a GDP-GTPγS exchange assay, performed on receptors expressed in insect cell membranes using the baculovirus expression system. The *E coli* and insect cell expression systems both produce NTS1 with equivalent neurotensin binding activities, but the *E. coli* system requires the presence of N- and C-terminal fusion proteins (MBP and TrxA, respectively) for high-level expression. As TrxA at the C-terminus could affect G protein coupling, untagged NTS1 and NTS1-7m were therefore expressed in insect cells for the G protein coupling assays. Although robust agonist-induced nucleotide exchange at Gαq was seen for wt-NTS1, only poor coupling was observed for NTS1-7m (Fig. 8B).

## Discussion

The rat neurotensin receptor NTS1 was an obvious target for thermostabilisation because NTS1 is not very stable in detergent solution, especially in short-chain detergents potentially useful for 3D crystallisation. Heterologous expression of NTS1 in *E. coli* and purification of functional receptors have been well established by Grisshammer *et al.*^30^. An essential step during the purification of NTS1 is the use of a neurotensin ligand-affinity column, which allows the enrichment of functional receptors. The application of NTS1 onto the NT column requires that no ligand is present at this time and, therefore, this dictated that NTS1 should be stabilised in the unliganded state to improve its stability during the steps before binding to the NT column. As crystallisation would likely involve the co-crystallisation of NTS1 with NT, it was also desirable that stabilised NTS1 would be at least equally stable in the presence of bound agonist as in the unliganded state. These two criteria governed the approach for the stabilisation procedure.

The identification and combination of thermostabilising point-mutations was performed by an Ala/Leu scanning methodology that had previously been used to stabilise both the β_1_AR and A_2a_R. Out of the best 31 thermostabilising point mutations identified in NTS1 by the −NT and +NT assays, the combination of the four mutations A86L, I260A, F342A and F358A produced one of the most thermostable mutants developed so far, NTS1-7m. NTS1-7m displayed several modified properties compared to wt-NTS1 including: an increase in thermostability of the solubilised, unpurified form (Fig. 5) and of the partially purified form as well as increase in thermostability in short-chain detergents (Fig. 7); a decrease in the NT dissociation rate (yet similar apparent NT affinity) and a smaller effect of Na^+^-induced NT dissociation (Fig. 8A); a decrease in antagonist affinity (Fig. 6); and reduced ability to functionally couple to Gαqβ_1_γ_1_ (Fig. 8B). Whilst it would be convenient to associate each of these properties with a single mutation within the receptor, it is likely that the combined effect of all the 4 mutations in NTS1-7m generates the overall characteristics of the mutant. For the purpose of the discussion, however, each property of NTS1-7m will be discussed in terms of how individual and/or combined mutations within NTS1-7m may contribute to its observed characteristics.

**Figure 9:**
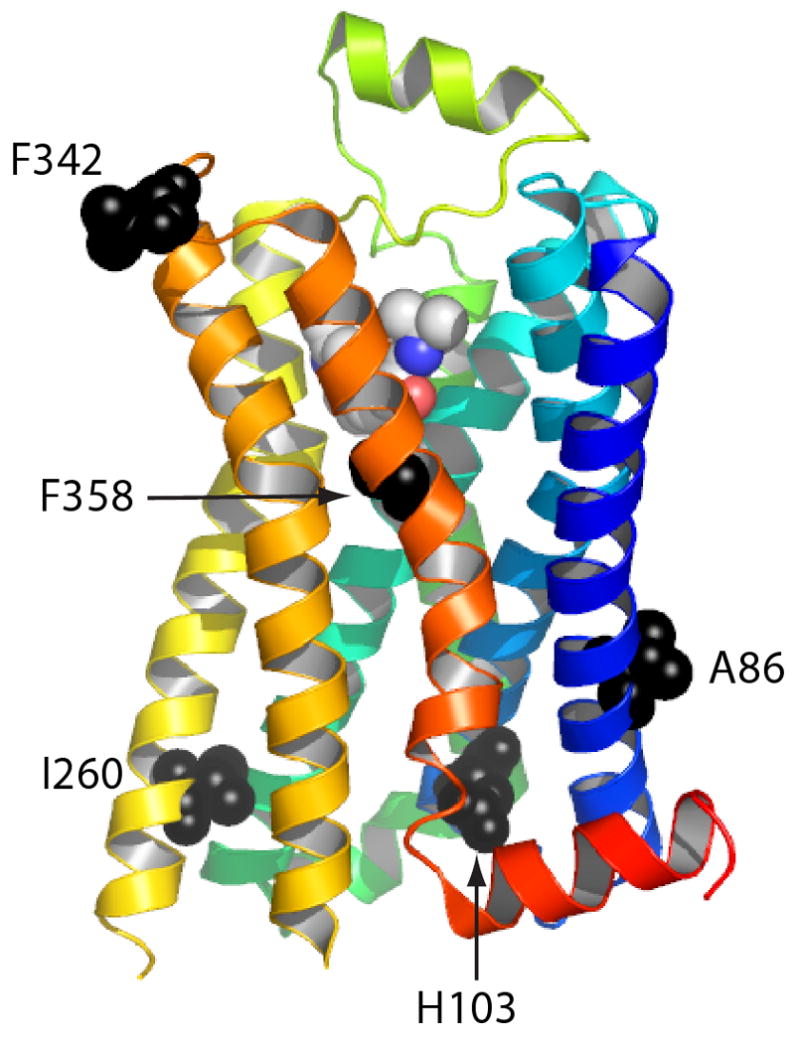
Positions of the thermostabilising mutations in NTS1-7m. The structure of the β_1_-adrenergic receptor (PDB 2vt4) is shown in rainbow coloration (N-terminus in blue, C-terminus in red) with the bound antagonist cyanopindolol shown as a space-filling model. The equivalent positions (via primary amino-acid sequence alignment) of five thermostabilising mutations of NTS1 are shown with the side chains as space-filling models (black) with the labels corresponding the amino acid residues in NTS1.

Several 3D structural models were made by overlaying the position of NTS1 amino acid primary sequence to bovine rhodopsin and β_1_AR-m23 crystal structures according to amino acid sequence alignment (Fig. 9, shown on the β_1_AR-m23 structure). The residue A86 is located in TM1; the equivalent amino acid side chains in the structures of β_2_AR (I55)^12^, β_1_AR (I63)^15^, rhodopsin (L59)^8^; ^9^ and A_2a_R (C28)^14^ all make contact with the lipid environment, but also with TM2. Based on the above receptor structures, the I260 side chain in TM5 is predicted to point towards the (D/E)RY motif in TM3. F342 is located in the extracellular loop 3 (ECL3) and is likely to be able to interact with residues known to form the ligand binding pocket. F358 is in TM7 and may interact with a conserved tryptophan residue in TM6 (position 6.48^36^) which constitutes the “toggle switch” for receptor activation^37^.

Binding of NT is clearly one of the important factors governing the stability of NTS1, even for the wt receptor. NT binding to the thermostable mutant NTS1-7m differed in several ways from that to wt-NTS1, although the K_D_s for binding were similar. The rate of dissociation of NT from NTS1-7m was 8-fold slower than for wt-NTS1. However, the rate of NT dissociation was only affected about 2-fold by the presence of 1M NaCl, compared to an acceleration of dissociation by over 50-fold for wt-NTS1. In contrast, the affinity of the antagonist SR142948 to NTS1-7m was reduced by ∼4-fold. The binding site for NT was predicted to include the region of ECL3^27^; ^38^; ^39^; thus the F342A mutation of NTS1-7m may be of importance in regard to its modified agonist binding properties. Whilst F342 has not previously been reported as being directly involved in NT binding, the ECL3 location of F342 places it within the region of the predicted receptor agonist binding pocket and modelling studies have implicated F342 in NT binding through its contribution to the aromatic character of the ligand binding pocket^39^.

The binding of antagonists to NTS1-7m was consistently weaker compared to wt-NTS1, whether it was measured as a Ki value from competition binding experiments between SR142948 and [^3^H]-NT (4-fold weaker binding; Fig. 6) or by saturation binding assay using [^3^H]-SR48692 (15-fold weaker binding; not shown). Reduced affinity of antagonist binding has been previously observed for F358A mutated NTS1^40^. Mutational studies combined with binding assays using SR48692 and its analogues have predicted π-π interactions between the dimethoxyphenyl group of the ligand and F358 of the receptor. SR48692 has additional predicted contacts at M208, F331, R327, Y324, Y351, T354, Y359 of the receptor, none of which are mutated in NTS1-7m.

In addition to the observed decrease in the NT off-rate, binding of NT to NTS1-7m was also not modulated by Na^+^ in a manner similar to wt-NTS1 (Fig. 8A). Like other rhodopsin-like GPCRs, NTS1 affinity for its agonist is influenced by the presence of Na^+^ with a decrease in the apparent NT affinity being observed with increasing Na^+^ concentration^34^. The Na^+^ effect can be eliminated by the mutation of a highly conserved aspartic acid residue in TM2 to an uncharged residue (D113A)^34^. The equivalent residue D83 in bovine rhodopsin, for example, is involved in an extensive H-bond network suggested to contribute to the stability and function of this receptor^9^. How the Na^+^ effect has been largely abolished by the mutations in NTS1-7m, none of which lie in TM2 remains unclear, nor is it obvious whether the Na^+^ effect observed with NTS1-7m relates to the Na^+^ effect involving D113.

While our primary purpose was to thermostabilise the receptor in various detergents, it is interesting to determine whether the mutant NTS1-7m could assume the activated (R*) conformation to couple to G-proteins, either in the absence or presence of NT. To our surprise, NTS1-7m did not efficiently catalyse nucleotide exchange at Gαq (Fig. 8B), either in the absence or presence of NT, even though NT could clearly bind to the receptor. This was unexpected because one of the thermostabilising mutations was F358A, which was previously shown to promote constitutive activity of NTS1^41^, so our expectation was to observe some constitutive activity of NTS1-7m in addition to agonist-induced G-protein coupling activity. Disruption of the intrahelical salt bridge (“ionic lock”) of the conserved (D/E)RY motif in TM3 is important for GPCR activation, and mutations within this motif have been associated with constitutive activity of GPCRs^24^; ^42^. In this regard the I260A mutation, in combination with the F358A mutation, of NTR1-7m may be playing a part. Sequence alignment^36^ of rat NTS1 with turkey β_1_AR, human β_2_AR, human A_2a_R, and bovine rhodopsin places I260 of NTR1 at V230 (β_1_AR), V222 (β_2_AR), I200 (A_2a_R), and L226 (rhodopsin). These hydrophobic side chains point towards the main chain atoms of the (D/E)RY tyrosine in the crystal structures of these receptors (PDB ID 2VT4, 2RH1, 3EML, 1GZM). It may be that removal of the isoleucine side chain in the NTS1 mutation I260A in some way counteracts the expected constitutively activating effect of the F358A mutation, as well as the ability to assume agonist-induced activated conformations.

The four mutations in NTS1-7m have clearly had an effect on the global conformation of the receptor. This is evident from the improved thermostability of NTS1-7m and the inability of NT-bound NTS1-7m to couple to G proteins efficiently. Only one of the four thermostabilising mutations could possibly interact with NT, based upon current models, suggesting that the other three mutations in combination are affecting the pharmacology of NTS1-7m through indirect effects. In the absence of a crystal structure, we cannot definitively say what conformation NTS1-7m has, but, given the G protein coupling data, when NT is bound it is not an activated state of the receptor.

One aim of producing NTS1-7m was to provide a mutant that is suitable for purification by the sequential use of Ni^2+^-NTA resin and a NT column^30^, and that is more stable than wt-NTS1. While the stability of solubilised NTS1-7m was improved compared to wt-NTS1 (*i.e.* beneficial during the initial step of receptor purification), the use of the NT column was not successful. This is largely because the off-rate of NT from NTS1-7m in the presence of high NaCl concentrations is considerably slower than for wt-NTS1. NTS1-7m binds to a NT column, but it cannot be eluted using high concentration (*e.g.* 1 M) NaCl, and therefore the NT column step in its present form is no longer an effective tool for purification. If we were to continue using the established purification procedures, further work will be required on the mutagenesis effort to stabilise NTS1 with different sets of mutations. Otherwise, a new purification scheme would need to be developed before crystallisation can be attempted with NTS1-7m.

Recently, a study by Sarkar *et al.* was published describing the evolution of NTS1 for expression and stability^43^. This approach was based on random mutagenesis by error-prone PCR, expression of the mutant library in *E. coli*, and identification of the most highly expressing mutants by FACS after binding a fluorescent NT analogue. This procedure identified the mutant D03, which contains 9 mutations (H103D, H105Y, A161V, R167L, R213L, V234L, H305R, S362A, S417C) located in TM2, TM3, ECL2, TM6, TM7 and the C-terminus. The NTS1 mutant D03 expressed almost 10-fold better in *E. coli* than wt-NTS1 and it appeared 3-4 fold more stable than wt-NTS1 in detergent at 45 °C (based on re-plotting the data in Fig. 5 of reference^43^). This work assumed that there was a correlation between expression levels and stability, but mutagenesis work on β_1_AR^19^, A_2a_R^18^, and NTS1 reported here (Fig. 1) show that stability and expression levels are only weakly correlated. Because of this, our approach to select the best receptor construct was based on choosing the most stable mutants regardless of their expression levels. Indeed, we found NTS1-7m to be 10-fold more stable at 45 °C in detergent solution (in the absence of NT, Fig. 7), with a similar expression level (less than 2-fold improvement) to that of wt-NTS1. In addition, this stabilisation effect was achieved by having only 4 mutations in NTS1-7m compared with 9 mutations (excluding the silent mutations) in the mutant D03. The most stabilising conditions we observed for NTS1-7m were in the presence of bound NT, and under these conditions NTS1-7m was 39-fold more stable than wt-NTS1, but equivalent figures for the mutant D03 are not available. One interesting finding in the mutant D03 was that its NT binding is also insensitive to Na^+^ concentration. Unlike NTS1-7m, however, D03 does contain mutations in TM2 near D113, as well as residues in TM3, which may be in contact with D113. Again, without a crystal structure, it is difficult to suggest how this effect has arisen.

We have now applied the approach of conformational thermostabilisation successfully to three GPCRs, NTS1 (this work), A_2a_R^18^ and β_1_AR^19^. In all cases, the receptor is stabilised in a conformation that preferentially binds either agonist or antagonist, depending upon which ligand was used during the selection procedure. Currently there is insufficient data to predict which mutation may be thermostabilising, so an Ala/Leu scan coupled to thermostability assays is still the best way to proceed. Our data also show that the position of thermostabilising mutations is different for each receptor, so it is likely that the transferability of thermostability between distantly related receptors is low. After producing the thermostabilised β_1_AR mutant, the structure containing bound antagonist was determined to 2.7 Å resolution^15^. The intrinsic instability of agonist-bound wt-NTS1 compared to antagonist-bound wt-β_1_AR suggested that more effort is needed to achieve an optimally stabilized neurotensin receptor suitable for crystallisation. Our work presented here shows that a systematic mutagenesis approach can be used to evolve a receptor that is thermostable both in the presence and absence of ligand. In addition, this selection strategy gave rise to mutant receptors that bind the agonist NT preferentially over antagonist SR142948, although the combination of two selection pressures on stability has resulted in a mutant receptor that virtually does not couple to G proteins. A different approach will be required to stabilise the receptor in a fully activated state, which may require the selection of thermostable mutants in the presence of the relevant G proteins.

## Materials and Methods

### Materials

The tritiated agonist, [^3^H]-neurotensin {[^3^H]-NT: [3,11-tyrosyl-3,5-^3^H(N)]-pyroGlu-Leu-Tyr-Glu-Asn-Lys-Pro-Arg-Arg-Pro-Tyr-Ile-Leu} was purchased from Perkin Elmer. The tritiated antagonist, [Methoxy-^3^H]-SR48692 {SR48692: {2-[(1-(7-chloro-4-quinolinyl)-5-(2,6-dimethoxyphenyl)pyrazol-3-yl)carbonylamino]tricyclo (3.3.1.1.^3.7^)decan-2-carboxylic acid}} was purchased from Amersham Biosciences/GE Healthcare (discontinued November 2007). Unlabelled neurotensin was purchased from Sigma. Unlabelled antagonist SR142948 {2-[[[5-(2,6-Dimethoxyphenyl)-1-[4-[[[3-(dimethylamino)propyl] methylamino]carbonyl]-2-(1-methylethyl)phenyl]-1H-pyrazol-3-yl]carbonyl]amino]-tricyclo[3.3.1.1^3.7^]decan-2-carboxylic acid} was purchased from Tocris Bioscience; referred to as SR142948A in reference 44. Detergents were purchased from the following suppliers: n-dodecyl-β-D-maltopyranoside (DDM, Glycon or Anatrace), n-decyl-β-D-maltopyranoside (DM, Anatrace), n-nonyl-β-D-glycopyranoside (NG, Anatrace), 3-[(3-cholamidopyropyl)dimethylammonio]-1-propanesulfonate (CHAPS, Anatrace), and cholesteryl hemisuccinate Tris salt (CHS, Sigma or Anatrace). Detergent concentrations are given as percent w/v (g/100-ml solution).

### NTS1 constructs for expression in E. coli and in insect cells

Wild-type NTS1 (wt-NTS1) refers to the N-terminally truncated rat neurotensin type I receptor starting at Thr^43^. Wild-type or a mutant form of receptor was expressed in *E. coli* as a fusion protein, with the *E. coli* maltose-binding protein (MBP) preceding the receptor N-terminus, and a thioredoxin-decahistidine tag (TrxA-H_10_) following the receptor C-terminus^30^; ^45^. For the construction of recombinant baculoviruses, the cDNA sequences for wt-NTS1 and NTS1-7m were subcloned into the baculovirus transfer vector pFastBac1 (Invitrogen) without coding for the fusion partners on N-terminal or C-terminal (Met-NTS1_T43-Y424_).

### Site-directed mutagenesis

Three hundred and forty (340) mutations were introduced throughout NTS1 from amino acid residues Ile^61^ to Thr^400^, spanning all seven TM helices, the putative helix 8, three intracellular and three extracellular loops and the proximal half of the C-terminus including potential phosphorylation sites. Each amino acid residue was changed to alanine, unless it was already an alanine in the wt-NTS1 sequence, then it was changed to leucine. Mutants were created by PCR-based site-directed mutagenesis using the *E. coli* expression plasmid as the template and following the QuikChangeII methodology (Stratagene) but using KOD hot start polymerase (Novagen). The positions of helices were predicted by aligning the amino acid sequence of rNTS1 with those of three other type 1 GPCRs: bovine opsin, turkey β_1_-adrenergic receptor (β-AR1), and human adenosine A_2a_ receptor (A_2a_R), and superposing the alignment onto the known crystal structure of bovine rhodopsin (PDB accession number 1GZM)^9^. Individual clones were fully sequenced in the NTS1 coding region to ensure that only the desired mutation was present. Multiple mutations were introduced to the receptor by including up to four pairs (only in one case five pairs) of mutagenesis primers in a PCR reaction, using a template already containing one mutation.

### Expression of NTS1 in E. coli for screening of mutants

Expression of wt-NTS1 and NTS1 mutants was performed in *E. coli*^30^ with modifications. Cultures were grown in 50-ml of 2×TY supplemented with 100 μg/ml ampicillin and 0.2 % glucose in 250-ml Erlenmayer flasks at 37 °C with shaking to an OD_600_ = 0.5. After addition of 0.5 mM IPTG, the temperature was lowered to 22 °C and the cultures were incubated with shaking for another 24 hours. Cultures were harvested as 2-ml aliquots by centrifugation at 13,000 ×g for 1 minute, flash frozen in liquid nitrogen, and stored at -20 °C.

### Radioligand binding and thermostability assays

Agonist binding to detergent-solubilized receptors was performed with [^3^H]-neurotensin ([^3^H]-NT)^30^. The harvested *E. coli* cells expressing wt- or mutant-NTS1 were suspended in lysis buffer [50 mM Tris•HCl, pH 7.4, 200 mM NaCl, 30 % (v/v) glycerol], supplemented with protease inhibitor cocktail (Roche), 0.75 mg/ml lysozyme, 25 μg/ml DNase I, 6.25 mM MgCl_2_, 0.1 % BSA and 0.004 % bacitracin. Receptors were solubilised by adding 1 % DDM, 0.6 % CHAPS and 0.12 % CHS (final volume 500 μl). After centrifugation, the cleared lysate was used directly in ligand binding assays (LBAs) in the assay buffer (50 mM Tris•HCl, pH 7.4, 1 mM EDTA, 0.1 % BSA, 0.004 % bacitracin, 30 % glycerol) containing detergents (0.22 % DDM, 0.6 % CHAPS, 0.12 % CHS, final concentrations after addition of lysate) and 12 nM [^3^H]-NT. The total number of functional receptors was determined by incubating the receptor in the assay buffer at 4 °C for 1 hour in the presence of [^3^H]-NT (‘normal’ LBA). Non-specific binding of [^3^H]-NT was assessed by either determining [^3^H]-NT binding to wt-NTS1 in the presence of 4 μM unlabeled NT, and/or performing LBA using DH5α cells not expressing any receptors in the presence or absence of 4 μM unlabeled NT. The amount of functional wt- or mutant-NTS1 (*i.e.*, receptors retaining ligand binding) was determined by a ‘spin assay’. Receptor-ligand complex was separated from free radioligand by applying the assay mixture on a spin column (QS-QM minicolumns, formerly supplied by Perkin Elmer, presently by Fisher Scientific) packed with Sephadex G50 (GE Healthcare), pre-equilibrated in 50 mM Tris•HCl, pH 7.4, 1 mM EDTA, 0.1 % DDM. The receptor-ligand complex was eluted by centrifugation and was analyzed by liquid scintillation counting (Beckman LS 6000).

Thermal stability was determined in the absence (‘-NT’ assay) or presence (‘+NT’ assay) of [^3^H]-NT. In the -NT assay, neither the [^3^H]-labelled, nor unlabelled NT was present during the incubation at 24 °C for 30 minutes. Samples were cooled on ice for 5 minutes and then ligand was added ([^3^H]-NT or [^3^H]-NT/unlabelled NT), and the samples were incubated for additional 1 hour on ice. The amount of functional receptors after heating was determined by spin assay as described above. In the +NT assay, the 30-minute incubation at 37 °C was done in the presence of [^3^H]-NT or [^3^H]-NT/unlabelled-NT. Samples were cooled on ice, then incubated on ice in the cold room for additional 1 hour before spin assay.

Saturation binding experiments were performed in the presence of 0.15 nM to 20 nM [^3^H]-NT using detergent-solubilised receptors in LBA assay buffer. Non-specific binding of NT to the receptor was determined by including 4 μM unlabelled NT. The apparent K_D_ values were obtained by non-linear regression analysis using a one-site saturation binding with a ligand depletion model in Prism software (GraphPad).

Thermal denaturation curves were constructed by incubating the solubilised receptors in the assay buffer in the absence (-NT) or in the presence (+NT) of 12 nM [^3^H]-NT at eight different temperatures between 0 and 70 °C for 30 minutes. The samples were cooled on ice, and ligand added to ‘-NT’ assay mixtures. All the samples were subjected to spin assay after the 1-hour incubation on ice. A potential change in non-specific binding upon heating at extreme temperatures was initially tested by carrying out thermal denaturaion experiments including 4 μM unlabelled NT in the assays; no changes in non-specific binding were seen after incubation at high temperatures. Data were analysed by nonlinear regression using a Boltzmann sigmoidal model in the Prism software.

### Denaturation time course of NTS1

To compare the stability of wt- and mutant-NTS1, the rate of thermal inactivation was tested by the decrease in activity over period of time. NTS1 fusion proteins were solubilised and +/-NT assays were prepared (as in thermostability assays) in large batches. Assay mixtures were aliquoted into eight equal amount (∼130 μl per tube) and heated at 45 °C in the presence or absence of [^3^H]-NT. One tube was taken out of the incubator at 8 different time points: 0 (no incubation at 45 °C), 5, 10, 20, 30, 45, 60, and 120 minutes for -NT assays and 0, 15, 30, 45, 60, 90, 120, and 180 minutes for +NT assays, and was plunge cooled on ice for 5-10 minutes. The tritiated ligand [^3^H]-NT was added to -NT assay samples. Samples were then incubated on ice for additional 1 hour before spin assay. Solubilised receptors were also heated for 0 and 120 minutes (-NT), and 0 and 180 minutes (+NT) at 45 °C in the presence of 4 μM unlabelled NT. No changes were observed in non-specific binding after long incubation. Data were analysed by nonlinear regression using a one-phase exponential decay model in Prism software.

### Ligand binding assays using intact E. coli cells

The frozen aliquots of cells expressing wt- or mutant-NTS1 were thawed on ice, resuspended in 1-ml cold TEBB buffer (50 mM Tris•HCl, pH 7.4, 1 mM EDTA supplemented with 0.1 % (w/v) BSA and 0.004 % bacitracin). A 50-μl aliquot of cell mixture was used in a total volume of 500-μl assay mixture (TEBB buffer and no detergents) containing 1 pM to 10 nM [^3^H]-NT. Non-specific binding of radioligand to the receptor was determined by including 4 μM unlabeled NT. The assay mixtures were incubated on ice for 2 hours, and then applied to GF/B glass-fiber filters (Whatman), pre-treated with polyethylenimine. The filters were washed three times with ice-cold TE buffer, dried, and counted in a Beckmann LS 6000 scintillation counter. The apparent K_D_ values were obtained by nonlinear regression analysis using a one-site saturation binding with ligand depletion model in Prism software.

The binding of the antagonist SR142948 to wt- and mutant-NTS1s was determined using unlabelled antagonist SR142948 in a competition assay format. LBAs on intact *E. coli* cells were carried out at the [^3^H]-NT concentration of 5 nM in a presence of 10 pM to 10 μM unlabelled antagonist. Assay samples were incubated on ice for 2 hours, then the mixture was applied to GF/B filters as described above. Saturation binding experiments of the same NTS1 samples were carried out in parallel to determine the apparent K_D_ values. The apparent K_i_ values were determined by nonlinear regression analysis using a one-site competition model in Prism software.

### Small-scale partial purification of NTS1 for stability tests in different detergents

The pellet from 100-ml *E. coli* culture, expressing wt- or mutant-NTS1 was solubilized in 6-ml lysis buffer [50 mM Tris•HCl, pH 7.4, 200 mM NaCl, 30 % (v/v) glycerol] containing 1 % DDM, 0.6 % CHAPS and 0.12 % CHS. Five hundred microlitres (500 μl, 50:50 slurry) of Ni^2+^-NTA agarose (Qiagen), pre-treated with binding buffer [50 mM Tris•HCl, pH 7.4, 30 % (v/v) glycerol, 50 mM imidazole, 100 mM NaCl, 0.1 % DDM, 0.6 % CHAPS and 0.12 % CHS] was incubated with 1-ml cleared lysate in the cold room with constant mixing for 1 hour. The resin was washed once with 1-ml binding buffer, then twice with 1-ml wash buffer [50 mM Tris•HCl, pH 7.4, 30 % (v/v) glycerol, 50 mM imidazole, 100 mM NaCl, and one of the desired detergents or the detergent combination (0.1 % DDM/0.6 % CHAPS/0.12 % CHS, 0.03 % DDM, 0.1 % DM or 0.3 % NG)]. The receptor was eluted from Ni^2+^-NTA resin using an elution buffer [50 mM Tris•HCl, pH 7.4, 30 % (v/v) glycerol, 200 mM imidazole, 100 mM NaCl and one of the desired detergents or the detergent combination]. Samples were subjected to spin assays. The thermostability was determined from denaturation profiles of the receptors in the desired detergent.

### Dissociation of NT from detergent-solubilised NTS1

NTS1 fusion proteins were solubilised in a volume of 25-ml containing 5-gram of wet *E. coli* cell paste as described^30^. Receptors (0.7-0.8 nM) were incubated on ice for 2 hours with [^3^H]-NT (2 nM) in assay buffer (50 mM Tris•HCl, pH 7.4, 1 mM EDTA, 0.1 % BSA, 0.004 % bacitracin) containing detergent (0.1 % DDM, 0.2 % CHAPS, 0.04 % CHS). [^3^H]-NT dissociation was then initiated by addition of 50 μM unlabeled NT, or by addition of 50 μM NT and NaCl (833 mM). Samples were subjected to spin assays using Bio-Spin 30 Tris columns (Bio-Rad)^30^ after the following incubation times: 0.1, 0.5, 1, 2, 3, 4, 5, 22 hours for wt-NTS1 (+/- NaCl), and additional time points, 25 and 29 hours, for NTS1-7m (+/- NaCl). The data were analysed by nonlinear regression analysis using a one-phase exponential decay model in Prism software.

### Expression of NTS1 in insect cells and preparation of P2 membranes

N-terminally truncated receptors (Met-NTS1_T43-Y424_) (see section *NTS1 constructs for expression in E. coli and in insect cell*) were produced in *Trichoplusia ni* insect cells using the baculovirus expression system. Insect cells were infected at a multiplicity of infection of 5 and incubated for 48 hours at 21 °C before harvest.

NTS1-enriched membranes were obtained as a P2 fraction from the insect cells essentially as described^46^, using a solution of 10 mM MOPS, pH 7.5, 5 mM EGTA, 100 μM 4-(2-aminoethyl)benzenesulfonyl fluoride HCl (AEBSF) as lysis buffer. The P2 membranes were resuspended in lysis buffer containing 12 % (w/w) sucrose, snap-frozen in liquid nitrogen, and stored at -80 °C.

Prior to G-protein coupling assays, the P2 membranes were treated with urea to remove peripherally bound membrane proteins^47^; ^48^. The urea-stripped membranes were resuspended in 12 % (w/w) sucrose containing MOPS buffer (10 mM, pH 7.5), snap-frozen in liquid nitrogen, and stored at -80 °C. The receptor density in urea-washed P2 membranes was determined by [^3^H]-NT saturation binding analysis. The samples were incubated for 1 hour on ice in 0.5-ml assay buffer (50 mM Tris•HCl, pH 7.4, 1 mM EDTA, 0.1 % BSA, 0.004 % bacitracin). Non-specific [^3^H]-NT binding was determined in the presence of 2 μM unlabeled NT. Separation of bound from free ligand was achieved by rapid filtration through GF/B glass-fibre filters pre-treated with polyethylenimine.

### GDP/GTPγS exchange assay

Cephalopod Gαq was purified from dark-adapted retinas of *Sepia officinalis* as described^47^. The dimer complex of Gβ1 and Gγ_1_ was purified from bovine retina^49^. The receptor-catalyzed exchange of GDP for GTPγS on Gαq was determined by modification of previously described procedures^46^; ^48^. Reactions were carried out in 12 ×75 mm siliconised borosilicate glass test tubes in a total assay volume of 50 μl. Urea-washed insect cell membranes containing wt-NTS1 or NTS1-7m were added to G-protein (Gαq Gβ_1_γ_1_) on ice to give a total volume of 30 μl. A reaction contained either the agonist NT, or the non-peptide antagonist SR48692^50^, or neither. GDP/GTPγS exchange was initiated by the addition of 20 μl solution of [^35^S]-GTPγS (Perkin Elmer). The final concentrations of components in each reaction were: 50 mM MOPS pH 7.5, 1 mM EDTA, 100 mM NaCl, 1 mM DTT, 3 mM MgSO_4_, 0.3 % BSA, 1 μM GDP, 4 nM [^35^S]-GTPγS, 40 μM AppNHp, 0.4 mM CMP, 1 nM receptor, 100 nM Gαq, 500 nM Gβ_1_γ_1_; and 10 μM NT, or 40 μM SR48692, or no ligand. The reaction mixtures were incubated at 30 °C for 5 minutes. Reactions were terminated by addition of 2-ml ice-cold stop buffer (20 mM Tris•HCl, pH 8.0, 100 mM NaCl, 25 mM MgCl_2_). The entire volume of each sample was filtered over a nitrocellulose membrane on a vacuum manifold. Filters were then washed six times with 2-ml ice-cold stop buffer. The nitrocellulose membranes were dried overnight and the radioactivity was quantified by liquid scintillation in a Beckman LS 6500 scintillation counter.

## Supplementary Material

01

02
