# Supplementary material for: Thermostabilisation of the neurotensin receptor NTS1
Source: J Mol Biol. Author manuscript; Available in PMC 2010 Jul 10. (PMC2696590; doi:10.1016/j.jmb.2009.04.068)
Supplement: 01 [file NIHMS115419-supplement-01.doc]

**SI Table 1: Summary of expression levels and single-point thermostability assays for NTS1 mutants containing one mutation**

|  | Expression^a^ | -NT score^b^ | +NT score^c^ | Selection |
| --- | --- | --- | --- | --- |
| wt | 750-1000 | 50 | 50 |  |
| L72A% | 512 | 68 | 23 | - |
| A73L | 648 | 58 | 67 | + |
| A86L | 1346 | 82 | 118 | ± |
| A90L | 1049 | 59 | 74 | + |
| L95A* | 1008 | 54 | 47 | - |
| H103A | 252 | 85 | 76 | ± |
| S108A | 348 | 70 | 31 | - |
| S112A* | 785 | 58 | 46 | - |
| D113A% | 429 | 159 | 24 | - |
| I116A | 352 | 74 | 39 | - |
| V165A | 674 | 53 | 75 | + |
| T179A | 788 | 62 | 30 | - |
| M181A^%^ | 95 | 70 | 39 | - |
| R183A | 144 | 52 | 64 | + |
| F189A% | 344 | 63 | 40 | - |
| S191A | 606 | 49 | 40 | - |
| L205A | 264 | 69 | 29 | - |
| T207A% | 216 | 65 | 31 | - |
| I260A | 1088 | 58 | 78 | + |
| V268A | 1060 | 66 | 44 | - |
| G306A^#^ | 1044 | 48 | 80 | + |
| L308A | 965 | 51 | 70 | + |
| F342A | 873 | 52 | 88 | + |
| D345A% | 135 | 84 | 32 | - |
| A356L | 616 | 86 | 41 | - |
| F358A | 622 | 74 | 109 | ± |
| S362A | 1289 | 64 | 66 | + |
| N370A^#^ | 2528 | 72 | 74 | ± |
| A385L | 887 | 81 | 56 | - |
| C386A | 927 | 80 | 43 | - |
| R392A | 1310 | 69 | 54 | - |
| H393A | 1591 | 75 | 46 | - |
| K397A | 1459 | 77 | 37 | - |
| P399A | 930 | 68 | 41 | - |

^a^ Average of 2-3 expression trials. The range of expression is given for wt-NTS1, as wt was expressed as positive control each time.

^b^ Average of 2 experiments.

^c^ Although repeated many times later on, only the initial screening value is given.

% Not further used due to its marginal stability in +NT format, combined with either low expression or marginal stability in -NT format.

* False positive in the initial screening. The first screening gave a much better score in the - NT format.

^^ Although good expression and stability, these mutations did not appear often due to difficulties in the PCR reactions. Both N370A and G306A were used in mutant combinations to test if they enhance expression and/or stability. The effect was, unfortunately, not significant.
