# Supplementary material for: Thermostabilisation of the neurotensin receptor NTS1
Source: J Mol Biol. Author manuscript; Available in PMC 2010 Jul 10. (PMC2696590; doi:10.1016/j.jmb.2009.04.068)
Supplement: 02 [file NIHMS115419-supplement-02.doc]

**SI Table 2: Summary of expression levels and stability of NTS1 mutants containing multiple mutations**

**both in the absence and presence of the agonist neurotensin**

| Mutant | Mutations | Expression^*^ | -NT score^*^ | +NT score^*^ | T_m(+NT)_ °C^#^ | K_D_(NT) nM^†^ | K_i_(SR) nM^†^ |
| --- | --- | --- | --- | --- | --- | --- | --- |
| 6a | A86L + H103A | 17 | 29 | 91 | -- | -- | -- |
| 6b | A86L + I260A | 48 | 11 | 47 | -- | -- | -- |
| 6c | A86L + F342A | 50 | 23 | 51 | -- | -- | -- |
| 6e | A86L + H103A + I260A | 46 | 18 | 48 | -- | -- | -- |
| 6f | A86L + H103A + F342A | 16 | 24 | 150 | -- | -- | -- |
| 6h | A86L + I260A + F342A | 40 | 28 | 85 | 47±1 | -- | -- |
| 6k | A86L + H103A + I260A + F342A | 60 | 38 | 115 | -- | -- | -- |
| 7a | F358A + A86L | 24 | 20 | 77 | 48±1 | 0.10±0.02 | 0.58±0.04 |
| 7b | F358A + H103A | 16 | 10 | 38 | -- | -- | -- |
| 7c | F358A + I260A | 5 | 27 | 78 | -- | -- | -- |
| 7d | F358A + F342A | 6 | 15 | 52 | -- | -- | -- |
| 7e | F358A + A86L + H103A | 48 | 35 | 87 | 48±1 | 0.14±0.02 | 0.91±0.18 |
| 7f | F358A + A86L + I260A | 24 | 25 | 122 | 49±1 | 0.04±0.01 | 0.73±0.07 |
| 7g | F358A + A86L + F342A | 40 | 39 | 131 | 50±1 | 0.21±0.02 | 2.8 ±0.2 |
| 7h | F358A + H103A + I260A | 6 | 13 | 63 | -- | -- | -- |
| 7i | F358A + H103A + F342A | 36 | 18 | 45 | -- | -- | -- |
| 7j | F358A + I260A + F342A | 43 | 12 | 47 | -- | -- | -- |
| 7k | F358A + A86L + H103A + I260A | 86 | 35 | 123 | 49±1 | 0.32±0.03 | 0.81±0.41 |
| 7l | F358A + A86L + H103A + F342A | 86 | 48 | 116 | 50±1 | 0.93±0.03 | 0.32±0.03 |
| 7m | F358A + A86L + I260A + F342A | 33 | 34 | 126 | 50±2 | 0.08±0.02 | 1.16±0.14 |
| 7n | F358A + H103A + I260A + F342A | 33 | 22 | 66 | 50±1 | -- | -- |
| 7o | F358A + A86L + H103A + I260A + F342A | 88 | 45 | 124 | 50±1 | 0.03±0.01 | 0.22±0.02 |
| 7p | H103A + I260A | 42 | 6 | 2 | -- | -- | -- |
| 7q | H103A + F342A | 16 | 14 | 4 | -- | -- | -- |
| 7r | I260A + F342A | 77 | 4 | 3 | -- | -- | -- |
| 7s | H103A + I260A + F342A | 63 | 2 | 4 | -- | -- | -- |

^*^ All the mutants were expressed as MBP fusion proteins in *E. coli*. The stability of the mutants in the absence of NT was measured by heating the DDM-solubilised receptor in the assay buffer at 37°C for 30 minutes. The stability of mutants in the presence of NT was measured by heating the DDM-solubilised receptor in the assay buffer at 47°C for 30 minutes. The expression levels are normalised against A86L, whose expression score was arbitrarily set to 100%. The stability values are normalised against A86L, whose +/-NT stability scores were 20%. Most of the values reported here are the averages of more than two independent experiments.

^#^ Apparent T_m_ values are determined in the presence of NT by heating the detergent-solubilised receptors in the assay buffer at 8 different temperatures. The T_m_ values were determined by non-linear regression analysis using a single-site sigmoidal model. The values are the average of 2-6 separate experiments and errors are the SEM.

^†^ “SR” refers to SR142948. Both K_D_ and K_i_ values are determined using the same samples of receptors on the intact *E coli* cells from saturation binding curves and competition curves obtained side-by-side. K_D_ values reported here were used to determine K_i_ values in the competition binding studies. Average K_D_ values from independent experiments are approximately within the standard errors.
